# Relationship Between Vertebral Fractures, Bone Mineral Density, and Osteometabolic Profile in HIV and Hepatitis B and C-Infected Patients Treated With ART

**DOI:** 10.3389/fendo.2019.00302

**Published:** 2019-05-14

**Authors:** Elisa Dalla Grana, Fabio Rigo, Massimiliano Lanzafame, Emanuela Lattuada, Silvia Suardi, Monica Mottes, Maria Teresa Valenti, Luca Dalle Carbonare

**Affiliations:** ^1^Internal Medicine Section D, Department of Medicine, University of Verona, Verona, Italy; ^2^Department of Diagnostics and Public Health, University of Verona, Verona, Italy; ^3^Department of Neurosciences, Biomedicine and Movement Sciences, University of Verona, Verona, Italy

**Keywords:** bone, DXA, fractures, spine, HIV, turnover

## Abstract

**Objective:** The purpose of our study was to evaluate the alterations of bone metabolism and the prevalence of vertebral fractures in the population with HIV and hepatitis B and C seropositivity in treatment with antiretroviral drugs (HAART).

**Methods:** We selected 83 patients with diagnosis of HIV, HBV, HCV infection. In all these patients biochemical examinations of phospho-calcium metabolism and a densitometry of lumbar spine were performed. We also evaluated lateral spine X-rays in order to analyze the presence of vertebral deformities and to define their severity. As a control group we analyzed the prevalence of vertebral fractures in a group of 40 non-infectious patients.

**Results:** We selected 82 seropositive patients, 46 males and 37 females, with a median age of 55 ± 10 years. Out of these patients, 55 were infected by HIV, 12 were infected by HBV, 11 presented HIV and HCV co-infection and 4 were HCV+. The prevalence of hypovitaminosis D in the studied population was 53%, while the prevalence of osteoporosis and osteopenia was 14 and 48%, respectively. The average T-score in the fractured population was −1.9 SD. The viral load and the CD4+ cell count were respectively, directly, and inversely correlated with the number and severity of vertebral fractures. Antiretroviral therapy regimen containing TDF and PI was a significant determinant of the presence of vertebral deformities. The use of these drugs was also associated with lower levels of vitamin D and higher bone turnover levels compared to other antiretroviral drugs.

**Conclusions:** HIV patients suffer from bone fragility, particularly at spine, independently by the level of bone mineral density. In this population, the T-score threshold for the risk of fracture is higher than that usually used in general population. For this reason, it would be indicated to perform an X-ray of the spine in order to detect vertebral deformities even in patients with a normal or slighlty reduced bone mineral density.

## Introduction

The introduction of antiretroviral therapy (ART) has certainly improved the prognosis of HIV patients by reducing their mortality and morbidity, but has also revealed some complications related to the therapy. Among these, the evidence of a greater prevalence of reduced Bone Mineral Density (BMD), osteopenia and osteoporosis in the HIV+ population than the control population has emerged and has been widely studied (1–5).

An important meta-analysis of studies comparing BMD between HIV patients with normal population showed a prevalence of reduced BMD in 67% of people with HIV and a prevalence of 15% of osteoporosis in this population (6). A more recent study shows that even in newly diagnosed, therapy-naive HIV-infected patients, without any known secondary causes of osteoporosis, low BMD and high bone resorption are significantly prevalent (7).

The increased risk of osteopenia/osteoporosis in HIV+ patients is not only due to ART: in these patients there are ART-related, HIV-related, and even not-HIV not-ART-related risk factors for bone loss (8, 9). From this, it is clearly demonstrated that the pathogenesis of reduced bone mass is multifactorial. As a consequence of reduced BMD, patients with HIV infection are more likely to present with fragility fractures (10–14).

Nevertheless, data on the prevalence of fragility fractures in the HIV+ population are poor and often based on retrospective evaluation of clinical fractures, but this approach dramatically underestimates the phenomenon (14). Some studies report a moderate increase in prevalence of fractures in HIV+ patients compared to the general population, but data are statistically not significant considering that other risk factors for fractures, such as low BMI, smoking, and alcohol abuse, show a high prevalence in these patients (12–16). Other studies, however, show a significant increased prevalence of fractures, especially in relation to the high prevalence of frailty (10%, about twice as much as in the general population) and increased propensity to fall (16). Comparison of fracture prevalence rates among HIV+ individuals and not-infected individuals in a large US healthcare system reported a significantly high prevalence of all fractures in HIV+ males and females (2.87 per 100 infected persons vs. 1.77 per 100 people per year in not-infected persons) in all venues, but above all to the proximal femur, spine and wrist (14). Subsequent US studies have confirmed the association between HIV and increased risk of fractures, including the Veteran Aging Cohort Study (VACS) and the US HIV Outpatient Study (HOPS) (17, 18). In patients with HIV and HCV co-infection, the incidence rate for all fractures (traumatic and by fragility) varies from 26.8 to 62.3 per 1,000 people per year, while the incidence of fragility fractures is 2.6 per 1,000 people per year (19, 20). Hepatitis C co-infection would be, according to these data, an independent risk factor for fractures along with other documented risk factors: cigarette smoking, alcohol abuse, advanced age, white race, low BMI.

According to two Italian studies, data on prevalence of fractures in the HIV-positive population would be even greater if we consider the vertebral deformities evaluable with a lateral spine X-Ray using a semi-quantitative evaluation of vertebral heights: according to Borderi et al. one HIV+ patient out of four would have a vertebral fracture and this data would be of potential clinical relevance, suggesting that bone damage is much more frequent than previously evaluated found by bone density evaluation (DXA) (21). The study of Torti et al. shows a prevalence of fractures in the male HIV+ population 2·5 times greater than a comparable age control group (22). A more recent study confirms that the prevalence of asymptomatic vertebral fractures is high in HIV-infected patients and it is not identified by the presence of osteoporosis in spine neither predicted by the FRAX equation (23). A low trabecular bone score (TBS) may be more associated with vertebral fractures than BMD, according with another recent Italian study (24).

The results of these studies, therefore, seem to indicate that lateral spine X-ray plays an important role in screening osteoporosis in this population, even before or independently from DXA. In fact, the measurement of bone mineral density alone cannot exclude the risk of fragility fractures, since many of these fractures occur in patients with normal BMD, as seen in other diseases, such as type 1 and 2 diabetes and acromegaly (25, 26).

The role of ART in increasing the incidence of fractures has not been completely established: up to now, data seems to converge on an increased incidence of fractures among HIV+ patients exposed to therapy than ART-naive patients, suggesting the role of certain antiretroviral agents, such as tenofovir disoproxil fumarate (TDF) and protease inhibitors (PI), in increasing the risk of fractures (27, 28). However, another recent case-control study does not support these data and suggests a reduction in the risk of fractures with prolonged exposure to tenofovir (29). In the light of this, further studies are needed to evaluate the effect of ART on fracture risk, especially for those patients who also present other risk factors.

The purpose of our study was to evaluate the alterations of bone metabolism and the prevalence of vertebral fractures in a population of patients with HIV and hepatitis B and C seropositivity in treatment with antiretroviral drugs.

## Methods

A cross-sectional study of 82 patients infected by HIV or hepatitis B and C was performed. We enrolled both HIV-infected population and hepatitis-infected population, in order to see if there were any difference in the phospho-calcium metabolism and in the prevalence of vertebral fractures between the two groups. To do so, we considered the two populations to be comparable both for the inflammatory background and for the antiretroviral therapy that both groups experienced. A database was created using data from clinical charts of HIV-infected patients referring to the skeletal metabolism outpatient clinic of the Day Hospital of Infectious Diseases and from patients of the Infectious Diseases Department of Policlinico G.B. Rossi in Verona from November 2015 to June 2017. Inclusion criteria in the study were: male and female patients over the age of 40 with a previous or new diagnosis of HIV and/or HBV seropositivity. A minority of patients also had coinfection from HIV and HCV. For every patients we considered the principal epidemiological, clinical, immunological and virological risk factors for osteoporosis: sex, age, race, weight, CD4+ cell count, HIV viral load, AIDS diagnosis, protease inhibitors and NNRTI exposure, hepatitis C co-infection, vitamin D deficiency, chronic renal insufficiency, menopause.

All patients with independent risk factors for impaired bone metabolism, such as concomitant diseases or infections, anticonvulsant intake or steroid therapy, kidney failure, and those who had taken drugs that could affect skeletal metabolism or bone mass were excluded from the study. Patients with severe hepatic disease (defined as severe increase in hepatic cytolysis indices, severe alteration of liver function indices or end-stage cirrhosis) were also excluded from the study. All the patients had been supplemented with a single monthly dosage of 100,000 IU of cholecalciferol before our study.

All patients enrolled in the study performed biochemical examinations for dosing the following parameters of phospho-calcium metabolism in serum: 25-OH vitamin D, Creatinine, Phosphorus (P), Calcium (Ca), Parathyroid hormone (PTH), C-terminal fragment of type I collagen (s-CTX). Moreover, calcium excretion was evaluated in urine. In 71 patients, bone densitometry was performed using DXA (Dual Energy X-Ray Absorptiometry). In all of patients the lumbar spine densitometry was performed, considering L1-L4 segment of the lumbar spine. Given the young age of the patient enrolled, we considered lumbar densitometry in order to identify the presence of fractures. Vertebral deformities were detected on lateral spine X-ray using a semi-quantitative evaluation of vertebral heights and quantitative morphometric analysis of centrally digitized images. Fractures have been identified by manual measurement of 3 vertebral heights (anterior-Ha, middle-Hm, and posterior-Hp) and height ratios have been calculated (Ha/Hp, Hm/Hp, Hp/Hp of the upper vertebrae, Hp/Hp of the lower vertebrae). According to the classification of Genant, we defined the grade of each vertebral fracture, as follows: a decrease of 20–25% has been considered as a mild deformity (grade 1), of 26–40% as a moderate one (grade 2) and of >40% as a severe one (grade 3) (30). For each patient, the Spine Deformity Index (SDI) was calculated by summing the grade of vertebral deformities: SDI > 1 is indicative of vertebral fracture according to its definition (reduction in vertebral high of at least 4 mm or of 20%). All the measures were performed by the same physician. For X-rays, intraobserver agreement were 92.8% (κ = 0.79).

We also analyzed the prevalence of vertebral fractures in a group of 40 patients referring to the Osteoporosis Clinic of Internal Medicine of Policlinico G.B. Rossi in Verona, without HIV, HBV, or HCV infection, comparable to the studied population by sex, age and average T-score value.

## Statistical Analysis

Data were expressed as the mean ± standard deviation. Difference between groups were assessed by T-student test for uncoupled data. Frequencies were compared using Chi-square test, with Fisher correction where appropriate. Logistic regression models were used in the statistical analysis of factors associated with the presence of vertebral fractures. ROC analysis was performed in order to evaluate the best cut-off of DXA expressed as T-score in terms of sensibility and specificity to predict the presence of vertebral fractures in this specific setting. Statistical significance was assumed when *P*-values were < 0.05. Statistical analysis was carried out using SPSS for Windows version 22.0 (SPSS Inc., Chicago, IL, USA).

## Results

We selected 82 seropositive patients, 46 males and 37 females, with a median age of 55 ± 10 years. The average duration of the disease from the time of diagnosis in the selected patients was 17 ± 10 years. Results of anthropometric, biochemical and densitometric parameters grouped by the presence of fractures are reported in Table 1. Note that the only significant difference between the two groups was the CD4 cell count which was lower in the fractured patients. Out of these patients, 55 were infected by HIV, 12 were infected by HBV, 11 presented HIV and HCV co-infection, and 4 were HCV+. Most of the HIV+ and HBV+ patients were experienced to antiretroviral therapy, as showed in Table 2. The median age of HIV-infected population was 54 ± 9 years and, of these, 29 were males and 26 were females. In HBV-infected patients the median age was 56 ± 11 years, 7 were males and 5 females. The median age of HIV/HCV-positive people was 51 ± 4 years, 9 males and 2 females. The last group of HCV+ patients was composed of 1 male and 3 females, whose median age was 63 ± 10 years.

**Table 1:** Characteristics of the studied population grouped by the presence of fractures.

**Parameter**	**Fractured mean ± SD**	**Not fractured mean ± SD**
Age (years)	57 ± 11	53 ± 8
Weight (kg)	66 ± 14	62 ± 12
Height (m)	1.65 ± 1.10	1.63 ± 1.10
Creatinine (mg/dL)	0.83 ± 0.24	0.80 ± 0.17
PTH (pg/mL)	33 ± 19	28 ± 17
Vit.D (ng/mL)	27 ± 7	34 ± 8
Calcium (mg/dL)	9.3 ± 1.2	9.3 ± 1.3
CTX (ng/mL)	0.52 ± 0.38	0.52 ± 0.29
Phosphorus (mg/dL)	3.1 ± 0.6	3.0 ± 0.4
BMD lumbar spine (g/cm^2^)	0.87 ± 0.11	0.91 ± 0.14
*T*-score of the lumbar spine	−1.9 ± 0.9	−1.5 ± 1.0
*Z*-score lumbar spine	−1.1 ± 1.0	−0.8 ± 1.2
CD 4 (cell/mm^3^)	477 ± 261^*^	720 ± 174

* *p < 0.001*.

**Table 2:** Distribution of ART in the population.

**Drug**	**Number of treated patients**
Tenofovir	36
PI	17
Tenofovir + PI	11
Other drugs	11
ART-naïve	8

No statistical differences in the main parameters of phospho-calcium metabolism have been detected between population infected by HIV and population infected by hepatitis viruses (HBV and HCV).

All of patients have been supplemented with cholecalciferol before our study with a single monthly dose of 100.000 IU; despite this, the median levels of vitamin D in the population were under the normal range (28.7 ± 14.4 ng/ml).

Analyzing data from densitometry, osteoporosis was present only in 17% of the population, osteopenia in 56.3%, while normal bone mass was present in 26.7% of the population. The prevalence of fractures was high: 34 patients presented at least one vertebral deformity (41% of the population). People with vertebral fractures were significantly older (>60 years old) and fractures were also more severe in older population than in younger population (*p* < 0.001). In the group of patients treated with bone-affecting drugs (tenofovir, protease inhibitors or a combination of them), the mean number of fractures was significantly higher than in the group who didn't receive these drugs (1.4 ± 0.9 vs. 0.7 ± 0.5, *p* < 0.05). Similarly, the median SDI (Spine Deformity Index) was higher in the first group than in the second one (2.2 ± 1.1 vs. 0.86 ± 1.00, *p* < 0.05). Of the fractured patients, 44% (15 patients) had grade 1 fractures, 41% (14 patients) had grade 2 fractures and 15% (5 patients) had grade 3 fractures. Multiple vertebral fractures were present in 25 patients (30.4%).

By the logistic regression considering the presence of fractures, the CD4+ cell count emerged as a predictive factor of the presence of fractures, after correction for bone mass, age, vitamin D levels and other parameters of phospho-calcium metabolism (Table 3). CD4+ cell count negatively correlated with the number of fractures and also with SDI (Spine Deformity Index) (*p* < 0.01 and R^2^ = −0.41). The viral load directly correlated with the presence and the severity of fractures (*p* < 0.001 and R^2^ < 0.45). It also directly correlated with PTH levels and negatively correlated with vitamin D levels. Furthermore, there was a negative correlation between CD4+ cell count and the viral load.

**Table 3:** Logistic regression evaluating the predictive factors of vertebral fractures.

**Variables in the equation**									
		**B**	**S.E**	**Wald**	**df**	**Sig**.	**Exp(B)**	**95% C.I. for Exp(B)**	
								**Lower**	**Upper**
STEP 1^a^	Age	−0.058	0.043	1.827	1	0.176	0.943	0.867	1.027
	CD4 cell	0.004	0.002	4.585	1	0.032	1.004	1.000	1.008
	Viralload	−0.002	0.008	0.086	1	0.769	0.998	0.982	1.014
	Creatinine	−2.006	1.951	1.056	1	0.304	0.135	0.003	6.165
	PTH	−0.012	0.030	0.157	1	0.692	0.988	0.932	1.048
	Vit.D	0.032	0.035	0.808	1	0.369	1.032	0.963	1.106
	Calcium	0.741	1.162	0.407	1	0.524	2.098	0.215	20.472
	BMD lumbar spine	2.279	8.244	0.076	1	0.782	9.764	0.000	101595310
	T-score lumbar spine	−0.194	0.870	0.049	1	0.824	0.824	0.150	4.536
	Constant	−7.493	12.375	0.367	1	0.545	0.001		

a *Variable(s) entered on step 1:age, CD4 cell, vialload, creatinine, PTH, vit.D, calcium, BMD lumbar spine, T-score lumbar spine*.

*Note that CD4 count was the main determinant after the correction for age, viral load, cretinine and the bone metabolism parameters*.

The mean T-score in patients with fractures was −1.9 ± 0.9 and the mean Z-score was −1.1 ± 1.2, while the mean T-score in patients without any fracture was −1.5 ± 0.8 without statistically significant differences between the two groups. Bone Mass Density (BMD) didn't correlate with the presence of fractures.

**Figure 1:**
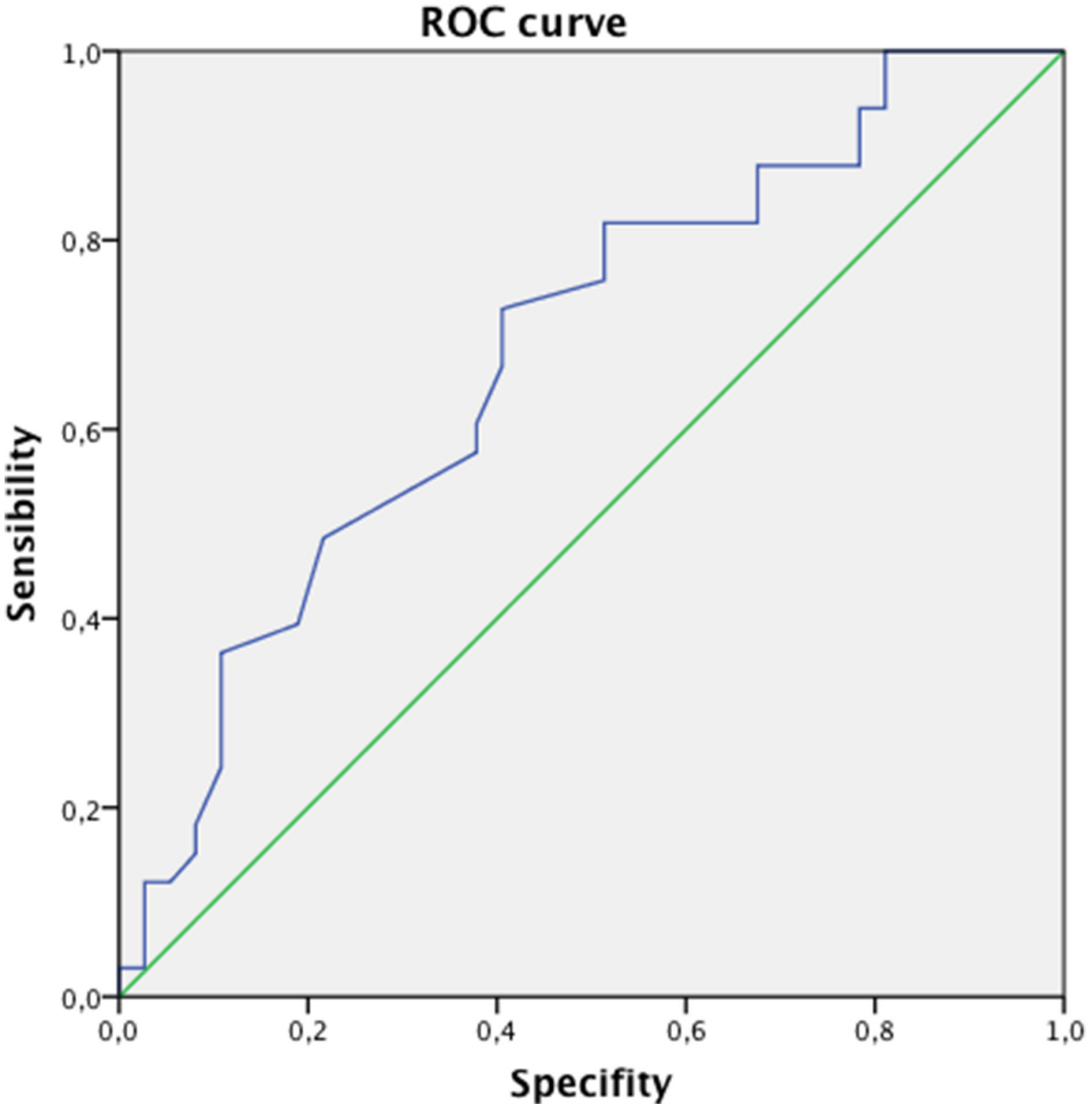
The ROC analysis between bone density and presence of vertebral fracture. The AUC for this diagnostic tool in this setting was 0.68 and the best cut-of was 1.7 SD showing 73% of sensitivity and 41% of specificity.

To better evaluate the relationship between bone density and presence of vertebral fracture in this specific setting, we performed ROC analysis using lumbar spine T-score as test variable. The AUC for this diagnostic tool in this setting was 0.68 and the best cut-off was 1.7 SD showing 73% of sensitivity and 41% of specificity with a Yourden's index of 0.32 (Figure 1).

**Figure 2:**
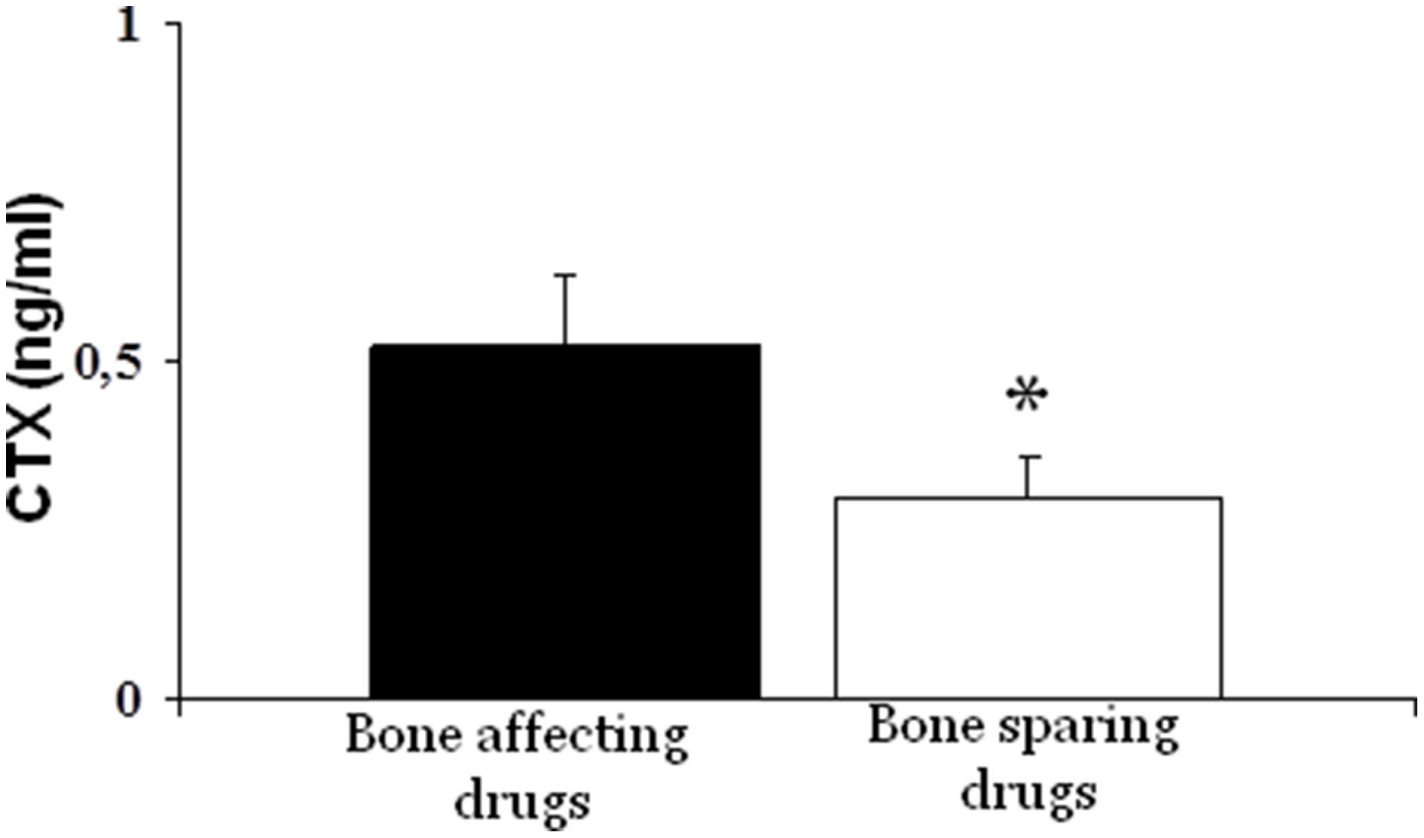
Bone resorption marker (CTX) was significantly higher in patients treated with bone-affecting drugs compared to bone-sparing drugs treated patients (*p < 0.001).

Analyzing the main parameters of phospho-calcium metabolism, resorption marker (sCTX) in the group of patients receiving drugs inducing bone loss was significantly higher than that of patients who did not receive these drugs (0.52 ± 0.31 vs. 0.30 ± 0.08, *p* < 0.001) (Figure 2).

**Figure 3:**
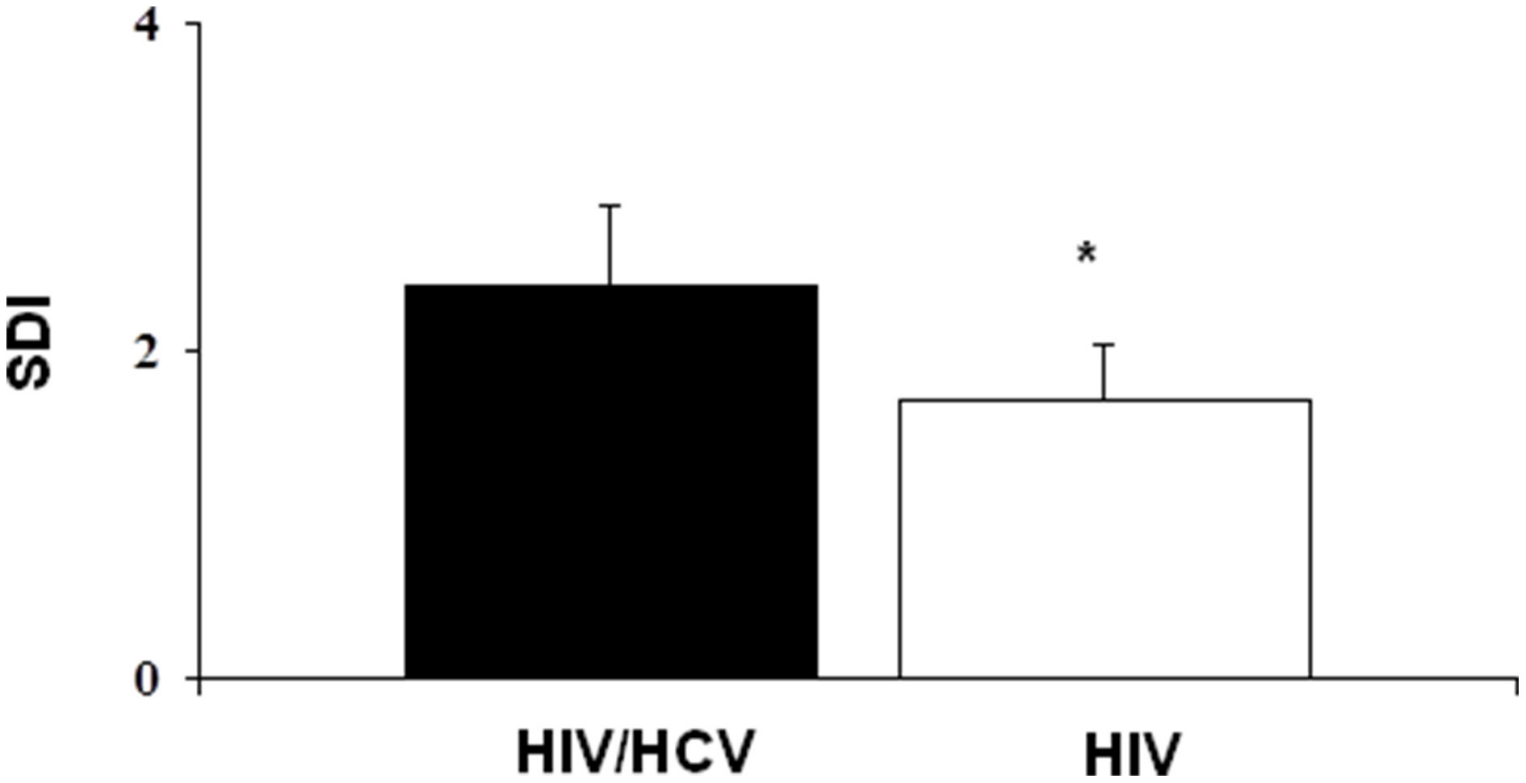
The severity of fractures was higher in HIV+HCV co-infected population compared to HIV-infected population (*p < 0.05).

Finally, comparing the population with HIV+HCV+ co-infection with the population with only HIV+ infection (Table 4), the mean T-score was significantly lower in the first group than in the second one (−2.2 ± 0.8 vs. −1.6 ± 1.2) (*p* < 0.05). Furthermore, fractures were more severe in the first group than in the second one, since SDI was significantly higher in the HIV+HCV+ co-infected population (2.4 ± 2.0 vs. 1.7 ± 1.0) (Figure 3). Most of the fractures (about 60%) were dorsal, with a wedge morphology and of grade 2.

**Table 4:** Differences between HIV and HIV + HCV population in parameters of phospho-calcic metabolism, features, and bone densitometry value.

	**HIV (*n*^**°**^55) mean±SD**	**HIV/HCV (*n*^**°**^11) mean ± SD**
Age (years)	54 ± 9	51 ± 4
PTH (pg/mL)	31 ± 14	24 ± 15
Vit. D (ng/mL)	28 ± 8	28 ± 10
Calcium (mg/dL)	9.3 ± 0.3	9.2 ± 0.4
CTX (ng/mL)	0.54 ± 0.34	0.36 ± 0.20
Phosphorus (mg/dL)	3.0 ± 0.5	3.1 ± 0.5
BMD lumbar spine (g/cm^2^)	0.91 ± 0.13	0.84 ± 0.10
*T*-score of the lumbar spine (SD)	−1.7 ± 0.2	−2.0 ± 0.8

We also analyzed the prevalence of vertebral fractures in a group of 40 patients referring to the Osteoporosis Clinic of Internal Medicine of Policlinico G.B. Rossi in Verona, without HIV, HBV or HCV infection, comparable to the studied population by sex, age and average T-score value. The main characteristics of the non-infectious group are described in Supplemental Table 1. The prevalence of fractures in these patients was significantly lower than that of the study population (17%).

## Discussion

Comparing the HIV-infected population and the hepatitis-infected population, no statistical differences were found in the main parameters of phosphocalcic metabolism, suggesting a similar bone disease in these patients.

Our study showed a very high prevalence of vertebral fractures in patients infected by HIV and hepatitis viruses: the 41% of the studied population showed at least one vertebral deformity. Despite the high prevalence of fractures, osteoporosis was present only in the 17% of the population enrolled in the study, while most of the population had a normal or a slightly reduced bone mass. In fact, the average T-score in fractured patients was −1.9 SD and the average Z-score −1.1 SD and these data were not statistically different from T-score measured in the non-fractured population (−1.5 SD). This means that bone mineral density could not be the best predictor of the risk of fragility fractures in these individuals and that a lateral X-rays at the spine could play an important role in detecting patients at a high risk of fracture even when DXA scan shows a normal BMD (22–24). In other words, these patients may experience vertebral fractures even when T-score levels are in the normal range. For this reason, in this population, it may be useful to define a threshold of fracture risk expressed in terms of T-score less negative than that traditionally considered for the general population. In our study, this T-score threshold was −1.7 SD, as expressed by ROC curve. In patients with T-score equal to or less than the proposed cut-off, it would be indicated to perform a radiographic study of the dorso-lumbar spine in latero-lateral projection in order to verify the presence of vertebral deformities and, where present, to define their severity.

In addition, bone mass levels correlated negatively with age: in older patients there were bone mineral density levels that were worse than in younger patients. The most plausible interpretation of this data might be that, on the one hand, age represents an independent risk factor for osteoporosis even in the general population, on the other hand, depending on the onset of the disease, elderly HIV patients may have suffered for more time the adverse effects of antiretroviral therapy, as many studies show (31–34).

Our study suggested that there could be some other predictors of risk fractures in this specific population, such as infection-related factors: CD4+ cell count and the viral load. Indeed, we found a direct correlation between the HIV viremia and the number and the severity of fractures and, on the other hand, an inverse correlation between CD4+ cell count and the number of fractures. These data confirm an increased fracture rate in individuals with worse indices of infection, as other studies have suggested (31, 32). HIV and Hepatitis C co-infection have consistently been reported to be associated with an increased fracture risk: in our study, the bone mass was significantly lower in individuals with HIV and HCV co-infection and the severity of fractures was, consequently, greater than that of the population with only HIV infection (20, 31). Hepatitis C co-infection was confirmed to be an independent predictor of incident fracture and this is probably explained by the effect of chronic liver disease present in these patients (19, 35).

Furthermore, we investigated the impact of antiretroviral therapy on the bone metabolism and also on the fracture risk. We compared the effect on bone metabolism of drugs recognized by the literature as mainly affecting the bone, i.e., tenofovir (TDF) and protease inhibitors (PI), with other drugs that did not appear to have this effect (32–35). We observed a significant increase in plasma CTX levels in the first group compared to the second group. Therefore, an antiretroviral therapy regimen that includes these drugs seems to increase bone resorption markers. Besides, patients in therapy with TDF and PI showed an increased fracture risk, since the average number of fractures as well as their degree of severity (SDI) were higher in this group of individuals than in the group who was not treated with such drugs.

Another interesting aspect in the use of antiretroviral therapy concerns the way it alters vitamin D metabolism lowering vitamin D levels in the blood (36–38) From the biochemical analyses of the patients, we found that the average levels of vitamin D were 28.7 ± 14.4 ng/ml and the prevalence of hypovitaminosis D was 53%, although all patients underwent supplementation with cholecalciferol before our study. Interestingly, a high viral load was associated with lower vitamin D levels: this could suggest that individuals with a worse progression of the disease have an impaired vitamin D metabolism. Furthermore, low levels of vitamin D in the blood have been correlated with an increased risk of vertebral fractures in HIV-infected patients, as some studies show (39). These findings, in line with what is reported in the literature, confirm the hypothesis that vitamin D metabolism is probably altered in these patients and that the normalization of 25-OH-D levels is achievable with higher doses of cholecalciferol than those usually used in the general population (40).

A limitation of the study is that this is a cross-sectional study, since we didn't take into account the duration of disease or even the duration of antiretroviral therapy in the patients considered. Furthermore, for some subgroups of patients, such as people with HIV and HCV coinfection or the HCV infected patients, the number of the population was significantly lower than the population of HIV infected patients. Another limit of the study is the lack of a control group. However, we compared the results from this study with those obtained from a population without HIV, HBV, HCV seropositivity matched by age, sex, and T-score value and we found that in these patients the prevalence of fractures was significantly lower than that of the studied population (17%).

## Conclusion

Adults with HIV, HBV, and HCV infection have an increased fracture risk compared to the general population age-matched, which is associated to an increased bone turnover due to the underlying disease and the antiretroviral therapy (ART). The role of antiretroviral treatment in genesis and maintenance of hypovitaminosis D and in the increase in skeletal turnover is also confirmed. A better control of the disease activity seems to be the first goal to reduce the impact on bone caused by the inflammatory background in these patients. On the other hand, the ART itself could lead to a worse outcome of bone metabolism as well as increased fracture risk.

The best approach to these patients should provide a detailed assessment of the parameters of bone metabolism including bone turnover markers. In addition, densitometry (DXA) is confirmed to be a useful tool for assessing possible bone mineral density impairment, although, in this particular setting, even moderately reduced bone mass levels may be associated with increased fracture risk. For this reason it should be necessary to define a different T-score threshold associated with an increased fracture risk. Consequently, in these patients it could be important to perform radiographs of the dorso-lumbar spine in order to detect vertebral fractures even in patients with a normal or slightly reduced bone mass.

The early detection of patients at a high risk of fractures allow to introduce a bone sparing treatment already in the first phases of the disease, by using wherever possible a low-impact antiretroviral therapy and by monitoring the presence of vertebral fractures.

## Ethics Statement

The study was carried out in accordance of normal clinical practice. No additional procedures were included in the study.

## Author Contributions

ED: patient selection and writing. FR: patient selection and data collection. ML: study design, data interpretation. EL: literature search, data collection. SS: patient selection and data collection. MM: study design, data interpretation. MV: study design, data collection. LD: data analysis, data interpretation, writing.

### Conflict of Interest Statement

The authors declare that the research was conducted in the absence of any commercial or financial relationships that could be construed as a potential conflict of interest.
