# Supplementary material for: Relationship Between Vertebral Fractures, Bone Mineral Density, and Osteometabolic Profile in HIV and Hepatitis B and C-Infected Patients Treated With ART
Source: Front Endocrinol (Lausanne). 2019 May 14;10:302. doi: 10.3389/fendo.2019.00302 (PMC6527878; doi:10.3389/fendo.2019.00302)
Supplement: Supplementary file 1 [file Table_1.docx]

| **Parameter** | **Mean** | **Standard deviation** |
| --- | --- | --- |
| Age (years) | 58,57 | 2,3 |
| Weight (kg) | 60,86 | 10,10 |
| Height (m) | 1,62 | 8,9 |
| Creatinine (mg/dl) | 0,87 | 0,42 |
| PTH (pg/dl) | 34,32 | 15,25 |
| Vit.D (ng/dl) | 34,70 | 9,20 |
| Calcium (mg/dl) | 9,51 | 0,21 |
| CTX (ng/ml) | 0,42 | 0,07 |
| Phosphorus (mg/ml) | 3,39 | 0,15 |
| BMD lumbar spine (g/cm^2^) | 0,91 | 0,06 |
| T-score of the lumbar spine (SD) | -2,04 | 0,26 |

**Supplemental table 1. Characteristics of the non infectious group (control group) . Note that the prevalence of vertebral fractures in this population was 17 %.**
